# Essays on Medical Education, No. III

**Published:** 1831

**Authors:** 


### WESTERN JOURNAL

OF THE

### MEDICAL AND PHYSICAL SCIENCES.

APRIL, MAY, AND JUNE, 1831.

### Essays and Cases.

Art. I. *Practical Essays on Medical Education and the Medical Profession, in the United States.*—By the Senior Editor.

### ESSAY III.

### MEDICAL COLLEGES.

Medical Colleges, on their present plan, were not known, till since the revival of letters, in Europe. For two or three centuries, they were few in number, and chiefly sustained by the genius and labors of individual professors, of whom Boerhaave, Haller, and the first Monro, may be cited as illustrious examples. The 18th century augmented their number, and established their importance; but it was reserved for the 19th to multiply them to an unprecedented degree, and show, that they may be composed in part, of men, whom the pride of science would, formerly, have pronounced unfit for such a lofty and difficult duty.

In the United States, the number at the close of the last century did not exceed three: it is now at least fifteen, and others are annually springing up. This extraordinary increase, may be ascribed, in part, to the great number of state sovereignties which make up our confederacy, each of which, instead of the federal government, grants college and university charters, and is ambitious to rival its neighbors in the number, if not the excellence of its institutions. But another cause is equally operative. This is the want of due care in the selection of professors; by which the standard of professional excellence is depressed to a level, that brings the office within the reach of unqualified aspirants, and offers to mediocrity of talents, a degree of encouragement which no age or nation ever before held out.

Did the best talent of the American profession find its way into our numerous schools, it cannot be doubted that they would be ably sustained; but truth and justice require me to say, that this is not always the case; and that every part of the union, presents men of loftier genius, sounder learning, and purer eloquence, than many of those, whom the trustees of our different institutions, from time to time, select as professors.

In most of our schools, the number of professors is too small. Some there are, or have been, which carry on all their operations, and graduate their pupils, with only four. Several have but five, and six are regarded as a *maximum*, which *need* never be exceeded. In Europe, however, the number is much greater; and in this respect we have unwisely departed from her example.

Nothing can be said in favor of this feature of our institutions. It has certainly not arisen from any new investigation of the principles on which medical schools should be established. It cannot be viewed but as the offspring of a competition, which has taken the downward, instead of the upward course, and offers a premium for pupils, in the small amount of its aggregate fees of tuition. Another aspect under which the same spirit manifests itself, is to be seen in certain schools, which have the ordinary number of professors, but charge little more than half the ordinary price for their tickets. Such institutions may be lauded as favoring the poor, but the real motive is, the acquisition of students, by the only means they are competent to wield.

For myself, I am convinced, that every medical school *ought* to have eight professors; and that with less than six, it should, in the phrase of Lord Bacon, 'be noted as defective.' As to the distribution of the duties, there may be some diversity of opinion; but the profession, generally, would perhaps concur in the following: 1. *Anatomy.* 2. *Institutes of Medicine.* 3. *Practice of Medicine and Clinical cases.* 4. *Surgery.* 5. *Materia Medica.* 6. *Chemistry and Pharmacy.* 7. *Obstetrics.* 8. *Medical Jurisprudence.* In addition to these branches, some of the professors, if qualified, should, in the summer, deliver lectures on the elements of Botany, Zoology and Comparative Anatomy, Mineralogy and Meteorology; all of which have relations with the medical profession, which will be perceived and appreciated, in proportion as juster and nobler views of its extent and dignity are disseminated. If but six professorships are established, Physiology should be united with Anatomy; the remainder of the Institutes with the Practice; and Medical Jurisprudence distributed among the chairs of Anatomy, Surgery, the Practice of Physic, Chemistry, and Obstetrics; an extent of association, which at once discloses the magnitude and complexity of this neglected branch of the profession, and evinces the necessities of confiding it to a separate professor.

In all the medical schools of the United States, the lectures of each preceding year, are, as to topics, the same with the last, and there is no classification of students, according to the different stages of their studies. Hence, it is not uncommon, to see a youth, in the first year of his studies, attempting to comprehend lectures on the Practice of Medicine, Operative Surgery, and even Practical Obstetrics, while, by his side will be seated, a candidate for graduation, attending to the first lessons in Descriptive Anatomy and Pharmaceutic Chemistry. There must be something wrong in a system, which leads to so great a sacrifice of time. I am not prepared to assert, that it would be practicable, to class our students of medicine into juniors and seniors, and have lectures adapted to each division, but I

would strongly recommend, to every student who visits a medical school, to direct his attention, chiefly, during the first course, upon Descriptive Anatomy, the Institutes of Medicine and of Surgery, Chemistry, Pharmacy, and the Natural History of Medicines; reserving, as the great objects of study for the second course, General and Surgical Anatomy, Operative Surgery, Therapeutic Materia Medica, Obstetrics, and tthe Diseases of Women and Children. In proportion as he observes this order, his time will be efficiently employed, and his knowledge well arranged.

A still greater evil than any of these, is the short sessions of our American schools. In some, the session continues but two or three months, and four are the prevailing term. The late celebrated Professor Wistar, admitted to me, in 1816, that the founders of the Philadelphia school, had committed a great error, in not establishing a session of six months, which would, doubtless, have been imitated by those who founded other institutions. The projector of the Medical College of Ohio, fixed its session at five months, which was, afterwards, unwisely reduced to four, although found to meet the approbation of the pupils. Were the different institutions of the United States, to agree to extend their sessions to five or six months, they would do more to elevate the profession, than could be done by any other single act; and all who wish well to the dignity of the profession and the interests of humanity, should unite in effecting so desirable an object.

By extending the term, the existing evil of so many lectures each day, would be obviated, and the student could find time to digest all that might be administered to him; as well as to compare what he should hear, with what he might see in the different text and standard works. Under existing circumstances, this is well known to be impracticable.

Four lectures a day, are as many as can be appropriated to itself, by an ordinary mind, and the number should never exceed five. But few students can listen to six, with as much advantage as they would derive from two-thirds of that number. They become cloyed and oppressed; and long before the end of the fourth month, find themselves fatigued, impatient, and irritable, and prepare to depart. I have no doubt, that thev would endure a session of six months, with fewer lectures, daily, in greater contentment, than they now bear our short sessions.

The daily succession of lectures, which requires the attention of the student to pass, rapidly, from subject to subject, which have no natural relations to each other, is trying to the intellect of pupils, and necessarily detracts much from the value of our instruction. For this evil, there is, perhaps, no practicable remedy; and students, generally, do not complain of it; as the change is refreshing, and serves to reconcile them to the wearisome existence of the lecture room.

Experiments and demonstrations are, or should be, the great object, in every medical institution. Much that is taught in lecture rooms, might be as successfully studied elsewhere; but Anatomy, Chemistry, Pharmacy, Operative Surgery, and Obstetrics, demand exhibitions to the eye, and no medical school is worthy of patronage, if deficient in the means of illustrating these important branches of the profession. Those, however, who endow and govern our institutions, should recollect, that the accumulation of the *materiel* of science, cannot be made a substitute for skill in its use; and that a department loaded with apparatus, will confer on students no substantial advantage, if the professor be defficient in talents and practical *tact.*

The subject of hospital practice, must not be passed over in silence. That it *might* be made a source of great improvement to students, no one can deny: that it *is* such, no one, I believe, will venture to assert. For a few weeks after the commencement of the course of lectures, the students are zealous in their attendance at the hospital, on prescribing days; bnt each one soon discovers, that in the throng, his opportunities for accurate personal observations, on the condition of the sick, are too few and momentary, to render his attendance in the wards of any substantial benefit; and his visits, thenceforward, become occasional and still more unproductive, because made under a feeling of discouragement. To derive benefit from hospital practice, students, who have the means, should attend upon it through the vacation, when the number is smaller, and the opportunities for personal observation, are, consequently, much greater.

Lecture room examinations are an important means of sustaining the attention of a class, but they are often misplaced, and sometimes worse than useless. The common practice of examining students, at the opening of a lecture, on the subject of the *preceding* day, is unfavorable, except when it serves to prepare the mind for the investigations on which the professor is about to enter. Whenever the topics to be discussed, are foreign to those of the last lecture, it should not be recalled, to preoccupy the attention of the students. Under such circumstances, a few questions on the matters which are to be immediately presented for examination, will prepare the minds of the pupils for engaging in them, and can never fail to awaken a spirit of inquiry. During a lecture, interrogatories on the subjects then before them, are well calculated to arouse the attention of the students from its passive condition, and contribute greatly to relieve the *tedium* of their situation. But it is at the close, that examinations are of the greatest value; as a recapitulation of the leading points, will, in this manner, be indelibly impressed on the mind.

Medical professors should be men of learning, but the lecture room is not the most proper place to display the extent of their book researches. The citation of a great number of authorities, is seldom profitable to students. They should expect from their teachers, an account, clearly and methodically expressed, of the ascertained facts of the science; and, in general, the less this account is encumbered with quotations and references, the better will it be understood, and the deeper will be the impression. Great learning may excite the admiration of students; but their thirst for useful knowledge can only be satisfied by accurate analysis, and the skilful arrangement, of common and generally admitted facts.

The abolition of the practice of publishing inaugural theses, seems to me to have been made inconsiderately. It has certainly destroyed one of the motives for close application, and while it has relieved the candidate from some trouble and expense, it unquestionably has diminished his exertions. It is well known, that when inaugural theses were printed, the desire to afford, in their first publication, an earnest of future distinction, led many students into extended courses of observation, research, or experiments; which served to awaken them to a proper estimate of their powers, and by committing them to the public as young men of learning or genius, gave an impulse of character, which often continued through all after life.

The rule that a student may matriculate at any time within the first month of the course, leads to great procrastination in starting from home; and in its bad effects is only equalled by the absurd custom of leaving the university before the expiration of the lectures. They cannot be too strongly reprobated. In all the schools of the United States, these customs prevail to a degree, that calls for an immediate corrective. But a single week should be allowed for matriculation after the introductory lectures are finished; and the student who leaves the school without permission, before the end of the lectures, should not be allowed credit for a course, when he proposes to graduate. In losing the two or three last weeks of the session, he does himself much injury, and disturbs the regular progress of those friends who have greater firmness of purpose, by turning their minds upon home and its endearments. The whole evil would be corrected, by requiring them to take of the Dean of the Faculty, a parting ticket, and I cannot but hope that this practice will be adopted in all the schools of the Union.

I come now to speak of things which depend more especially on the students themselves.

Many students commence attendance upon lectures, without due preparation. If they reside, or prosecute their studies from the beginning, in the neighborhood of medical schools, they may, with advantage, begin the study of Anatomy, Chemistry, and Pharmacy, by attending the lectures on those subjects. But little advantage can result from their commencing other branches of the profession, in the lecture room. From twelve to eighteen months of diligent reading is necessary, to prepare a student for listening with profit, and without confusion of mind, to lecturers on Surgery, the Institutes and Practice of Medicine, Materia Medica, and Obstetrics.

A timely entrance upon the course of the lectures, is scarcely less important, than a due preparation by previous studies. To avail himself of the full benefits of the session, a student should arrive and take comfortable lodgings, before the lectures commence. He thus brings his mind into a suitable condition for study, and, from the beginning, is able to derive advantage from what he hears.

Disorderly conduct and noise, in the lecture rooms, are great drawbacks on improvement. The idle and unambitious are, generally, the instigators of this confusion; which should be met and put down, by the public sentiment of the class, in conjunction with the moral power of the professor; neither of which is, in general, exerted in the manner it might be. Not a little of the disorder which lecture rooms present, arises from the practice of coming in after the lecture commences, and going out before it is finished. No member of the class should feel himself at liberty, thus to violate the laws of courtesy, and disturb the attention of those, who, unlike himself, are desirous of acquiring knowledge, the great end for which they are assembled.

Students should not only enter the lecture room in due time, but, as far as possible. return to the same seats, respectively; the advantage of which, both to pupil and professor, would be found in the uniform appearance which the room would present, from day to day, whereby the attention would not be distracted by new combinations and aspects of the class. Indeed, a return to the same seat is necessarily attended with an association of ideas, which is favorable to study, and, *cæteris paribus*, those who practise it most, will be most benefitted by the lectures.

The practice of cutting the benches, and otherwise marring, or eviscerating the room, should be punished, as a depredation, while the class should frown upon it, as puerile, and unfavorable to rapid improvement; which cannot fail to be more or less impeded, by the petty *violations*, which transform a beautiful and well ordered apartment, into a scene of dirt and desolation.

In short, students of medicine should be *gentlemen* in the lecture room, as well as the drawing room; and consider it discreditable, to abandon themselves to levity, mischief, and idleness; all of which are not less at variance with propriety than improvement.

Taking notes is an important employment of the lecture room, and one for which but few students are prepared. At the beginning of the course, most of them are zealous in this labour; but its difficulties, in many cases, speedily diminish this zeal, which is often succeeded by downright indifference. The pupil should be careful never to attempt too much, particularly during the first course. On Anatomy, Physiology, Chemistry, Pharmacy, Operative Surgery, and practical obstetrics, as a genral observation, notes should never be taken; and much of what constitutes courses of lectures on the institutes and practice of medicine, materia medica, the principles of surgery, the diseases of women and children, and medical jurisprudence, need not be recorded, because it may be found in the books. In the last century, when the practice of publishing every thing, did not prevail, as in the present age, the necessity of making notes was much greater. There is a value, however, in this labor, distinct from the manuscript information of which it puts the student in possession. It in some degree, secures and sustains his attention, and the extension and filling out of his notes, at his room, is a valuable exercise, leading to a kind of intellectual rumination, and favoring advancement in the important art of composition. Students, however, who can command their attention, and have retentive memories, would do well to write down but little, till they retire, when they should record such facts and principles, as appear to be original or of great practical utility. In the perception of these, more, perhaps, than in any thing else, will the discriminating or *instinctive* sagacity of the pupil, manifest itself. As students who write in the lecture room, can only record a part of what is said, they should exercise a quick, and, in loose phraseology, an intuitive judgment, as to what especially deserves to be recorded; otherwise, in laboring upon the common-place, the new and valuable may entirely escape their attention.

All the reading of the student, during the session, should have a reference to the lectures. He will find time for no other, if he properly study the great variety of topics, which will be daily presented to his attention. In the prosecution of these, reading will be of essential service, as connecting, in his mind, the information received through the two senses, and from at least two teachers—the lecturer and the author. They will be made to rectify and illustrate each other, and the curiosity to see how far the former is indebted to, or surpasses, the latter, will prove a beneficial stimulus.

Conversation and examining clubs are productive of much advantage, provided they are conducted with order—that is, by fixed laws: if not, they are worse than useless, as attracting the student from solitary study.

Practical anatomy should engage the attention of every pupil. In a country where all physicians must, to a greater or less extent, be operative surgeons, every candidate for the profession should learn the use of the scalpel; and work his way, for once at least, through the intricate fibres of the human body. Having seen, and with his own hands separated the parts from each other, their forms and relations will be well understood, and remembered for a much longer period. Moreover, unforeseen events, or the development of a new taste, may at length incite him, who was, originally, disinclined to the practice of surgery in its higher operations, to turn his attention to that elevated department of the profession, when he will find great difficulties to encounter, unless, while a student, he acquired a facility in the use of the knife and scissors, together with a sound knowledge of the anatomy of those parts, which are the chief seats of the greater operations.

As to the number of courses, I would say, that all students who have adequate pecuniary means, should attend three, before they graduate; and the second, if convenient, should be in a different institution, from the first and third, which should be in the same. I know from personal observation, that a great number of students graduate, prematurely; that is, without the extent and maturity of knowledge, which fits them for safe and efficient practice. It is a point of honor to graduate at the end of the second course, but the ambition of a student *should* be, to graduate *with honor*, rather than within a given time. Relaxation in elementary studies, is, commonly, the first effect of graduation; and he who is imperfect then, generally remains imperfect throughout life.

Almost all candidates have more or less perplexity on the subject of their inaugural dissertations. This arises in a great degree from delay. The summer preceding the last course of lectures, is the time, when the thesis ought to be prepared; but the majority postpone it till the midst of the second session, when its composition is attended with many difficulties; and sometimes keeps the candidate from the lecture room for several days, when his anxiety to be in regular attendance and prepare for his examination, is, generally, greater than at any preceding period. Such are the unsavoury fruits of procrastination.

Finally, students when in attendance on lectures, should look carefully to the preservation of their health. The new causes which may impair it, are, first, a more inactive and sedentary life; secondly, a fuller and more stimulating diet, than most of them have been accustomed to at home; thirdly, a change in the hours of eating, especially, breakfast and dinner, both of them being too late—the former, for an obvious reason, the latter, because, the appetite becomes importunate, and leads to gastric repletion; fourthly, in many cases abridgment of the hours of sleep; fifthly, intense and perplexing application of mind; sixthly, crowded and badly ventilated lodging rooms; finally, dissipated pleasures, into which they are too often seduced by the allurements of great cities, the common and most proper sites for medical institutions. From the operation of these, and other causes, the health of the medical students, during the session, is exceedingly apt to become impaired. The maladies to which they are the most liable are constipation, hepatic torpor and engorgement, dyspepsia, headache, chronic ophthalmia, palpitation of the heart, hypochondraism, *ennui*, jactitation, fidgets, and other forms of nervous irritation. With any of these maladies upon him, the student will make but sorry progress; most of them, however, might be prevented, by temperance, exercise in the open air, and general regularity of life and conduct.

I shall here present a summary view of the *desiderata* which our medical institutions seem to me to present; and can offer no other apology for the repetition it may involve, than the great importance of the subject.

1. An increase of the length of our sessions to at least five months. This could readily be effected, by a simultaneous resolution of the more popular and frequented institutions, in which our venerable *Alma Mater*, the university of Pennsylvania, ought, in justice to her age and relative rank, to take the lead.

2. An augmentation of the number of professors to at least seven, with a general refusal to recognize schools that have not more than four or five.

3. The election of professors for five or seven years, instead of an indefinite time; with the general understanding, that a re-election would in all cases take place, if the incumbent had acquitted himself with zeal and competent ability. If pupils were not compelled to take all the tickets twice, dull and unskilful professors would be neglected, and might at length, resign. But, as in nearly all our schools, the pupils are required, at each session, to purchase the whole of the tickets, the strong professors are made to sustain the weak, who of course do not resign, but clog the institution, impede its progress, and depress the standard of excellence. Septennial elections might prove a corrective to these evils, though I am not prepared to say, that a better could not be devised.

4. Students should not be allowed credit for a course, unless they matriculated by the end of the first week of the didactic lectures, and then remained to the end of the session.

5. At least four years should elapse, from the commencement of the pupil's studies, until his graduation.

6. He should be required to show that he is twenty-one years old.

7. Increased attention should be paid to the preparatory or academical attainments of candidates.

8. The examinations for a degree should be more searching than they are generally made. This, it is true, would diminish the amount of graduation fees received by professors, but the public would be gainers.

9. Every candidate should be required to publish his thesis; and a premium should be awarded to the author of the ablest of these productions.

10. Summer lectures, especially on the collateral and auxiliary sciences, ought to be encouraged, and the candidate should be required to have some knowledge of those branches.

11. A stricter regard should be had to the character of candidates, who should never be admitted to examination until they had deposited with the dean, satisfactory evidences of good reputation.

12. Lastly, as a means of promoting this object, and of advancing the respectability of the profession, there should, in every medical school, be a series of Sunday morning discourses, by the professors, on the *morale* of the profession, and the virtues and vices of Medical men, embracing their duties to their patients, and a system of medical ethics.

A strict attention to these different points, could not fail to improve the condition of our schools, and elevate the character of the American profession.

I shall conclude with a few hints to professors, on the means of making their prelections interesting and instructive.

A dull lecture is a great evil. Politeness may reconcile the majority of a class to such a lecture, but it falls dead-born from the lips of the professor. To listen, day after day, for several hours, through four months, even to animated speakers, is a serious undertaking; but to sit, from hour to hour, beneath those, who,

'Through the long, heavy, painful page, drawl on,'

is intolerable to all, who have not a facility in resorting to early and sound sleep; the usual and best resource, under such a calamity. As instruction is the object of all medical lectures, the end for which they were instituted will not be attained, if the attention of the class be not kept in activity. The moment it flags, improvement is at an end, and the professor had better fold up his manuscript, and permit the pupils to return to their chambers. There is an eloquence of the lecture room, as well as of the Bar and Pulpit, which every professor should attain, or feel himself in duty bound to resign, so as not to exclude a competent man. A commanding knowledge of the subject, is an indispensable prerequisite; but earnestness and animation of manner, are of equal importance; for without them, the profoundest learning and the acutest logic, are of no avail. At first, they may fix the attention, but, unaided by the arts of oratory, their power is soon lost, even upon the inquisitive and the resolute; while the remainder, *pars major*, neither hear nor think.

The faculty of awakening and sustaining the attention of an audience, is, in some degree, a gift of nature, and may be wanting, when other requisites are not. An original or eccentric manner, is often the secret of success; illustration by means of anecdotes, skilfully introduced, produces the same effect; episodes may be so managed as to answer the purpose; flights of fancy, if well timed, will accomplish the end in view; while, in the absence of a talent for the whole of these, unexpected and pertinent questions, with familiar, conversational remarks, on the answers that may be given, will resuscitate the drooping energies of the class, and enable them to hold out to the end. Professors who speak *ex tempore*, have, in this respect, a great advantage over those who read manuscripts, for the speaker *must* think, while the reader *need* not; and the class will generally follow their example. It has been objected to extemporary lectures, that they are apt to be repetitious, and deficient in method. Precision and arrangement, however, depend more on the mind of the teacher, than the circumstances under which his lectures are delivered. But in sciences so imperfect, as most of those taught in our medical schools, a natural arrangement is not practicable; and extreme attention to method, is often prejudicial, as it leads to assumptions, and the introduction of dubious facts, to eke out an argument, which, in the present state of the profession, should be left defective. Written lectures, moreover, are not easily altered, and, when once finished, are apt to be kept nearly in their original condition. Thus, what might have been as perfect, at first, as the state of the science permitted, is soon left behind, in the march of improvement; while the annual repetition of the same *formula* of words, diminishes the simplicity and force with which it is pronounced;

> 'And every year grows duller than the last.'

